# Emergence, identification, and characterization of a novel *Streptococcus agalactiae* CPS type Ia ST7 strain (Ia-2021) causing large scale mortalities in tilapia aquaculture

**DOI:** 10.1038/s41598-026-52208-0

**Published:** 2026-05-09

**Authors:** Benjamin R. LaFrentz, Paola Barato, William R. Keleher, Amber E. Johnston, Megan Raftery, Jason W. Abernathy

**Affiliations:** 1 United States Department of Agriculture – Agricultural Research Service, (USDA-ARS), Aquatic Animal Health Research Unit, Auburn, Alabama 36832 USA; 2 CORPAVET and MolecularVet SAS, Calle 24 # 3 – 27, Neiva, Huila Colombia; 3 MolecularVet US LLC, Davie, Florida 33330 USA; 4 Kennebec River Biosciences, Richmond, ME 04357 USA; 5 Present address: Aquaculture Research Institute, University of Maine, Orono, ME 04469 USA

**Keywords:** *Streptococcus agalactiae*, Streptococcosis, Capsular type, PCR assay, Typing, Whole genome phylogeny, *Srr-1*, Serine-rich repeat protein, Tilapia, Aquaculture, Diseases, Genetics, Microbiology

## Abstract

In 2021 streptococcosis caused by *Streptococcus agalactiae* capsular polysaccharide (CPS) type Ia ST7 (referred to as Ia-2021) emerged in Mexico and spread to other regions in Central and South America devastating the industries in the affected countries. To address this emergent issue, the present research aimed to: (1) document case reports of the disease, (2) reproduce clinical signs under laboratory conditions, (3) use comparative genomics to investigate the evolutionary history of the emergent strain, and (4) develop an assay to discriminate from other *S. agalactiae*. Experimental injection challenges recapitulated clinical signs and gross lesions of disease observed in the field including erratic swimming, external hemorrhages, pale livers, hemorrhaging and necrosis of the intestine, and pustules. Whole genome sequencing, comparative bacterial genomics, and phylogenetic analyses yielded a DNA target suitable for a discriminatory PCR assay, which reliably differentiated the CPS type Ia-2021 isolates from other CPS type Ia isolates. These new primers were included in a previously established multiplex PCR (mPCR) for *S. agalactiae* CPS typing. The validated mPCR provides a “one-stop shop” for *S. agalactiae* CPS typing and can reliably assign unknown isolates to one of the ten CPS types, as well as discriminate between the Ia-2021 strain from other Ia isolates. This assay fills a critical need in epidemiological surveillance in tilapia aquaculture and provides an important tool to ensure appropriate CPS types are incorporated into vaccines. The work presented here is a critical first step to accelerate research to address this emergent disease and develop practical solutions for tilapia farmers to prevent streptococcosis caused by *S. agalactiae* CPS type Ia-2021.

**Supplementary Information:**

The online version contains supplementary material available at 10.1038/s41598-026-52208-0.

## Introduction

Tilapia (*Oreochromis* spp.) are an important aquaculture species for food security and social/economic development. Tilapia ranks third in global finfish aquaculture production and in 2022 the worldwide production of Nile tilapia (*O. niloticus*) was estimated at 5.3 million tonnes^1^. In addition to providing a source of high quality protein, there is potential for increasing the use of processing by-products for food and other applications^2^. Tilapia aquaculture also generates employment opportunities and income in many countries, resulting in improved livelihoods and economic and social development^3^.

As with any animal production industry, disease threatens the sustainability of the industry. Tilapia are susceptible to an array of bacterial, viral, and parasitic pathogens^4–6^. Of the bacterial pathogens, the gram-positive *Streptococcus agalactiae* (group B Streptococcus, GBS), is the most prevalent and important species impacting global tilapia aquaculture. Clinical signs and gross lesions of streptococcosis in tilapia include loss of appetite, spiral or erratic swimming behaviour, skin haemorrhages, exophthalmia, corneal opacity, enlarged spleen, kidney and liver, and ascites in the abdomen^6,7^. Epizootics of *S. agalactiae* typically occur during the grow out phase of production when tilapia are greater than 50 g, but is more common in larger tilapia with sizes greater than 200 g through market size^8^. Prevention and control options include optimized husbandry and management strategies, biosecurity, vaccines, probiotics and functional feed additives, selective breeding, and antibiotics^9^.

To understand the population of *S. agalactiae* circulating in tilapia aquaculture, molecular epidemiological tools used for human and other animal *S. agalactiae* isolates have been applied. Two commonly used methods include capsular polysaccharide (CPS) typing and multilocus sequence typing (MLST). To date, ten CPS types have been described^10^. CPS types can be determined by agglutination assays using antisera specific for each CPS type, whole genome sequencing^11^, or molecular assays. The latter are based upon the presence of unique genes or specific sequences in the CPS biosynthesis operon that are specific to each CPS type. The gold standard for CPS typing by polymerase chain reaction (PCR) is the multiplex PCR (mPCR) developed by Imperi et al.^12^, which can readily assign an unknown isolate to one of the ten CPS types based upon the number and size of amplicons generated.

MLST is a typing technique based upon the presence of alleles in DNA sequences of housekeeping genes. Unknown isolates are assigned to sequence types (ST) based upon the profile of identified alleles in the target genes, and isolates can be assigned to clonal complexes (CC) which are groups of closely related isolates^13,14^. A MLST scheme based on seven loci was devised for *S. agalactiae*^15^ and has been used extensively to type isolates from various sources across the globe. A public database of these genotypes is available at PubMLST^16^.

In tilapia aquaculture, CPS types Ia, Ib, and III are the most prevalent across the global industry, but there are regional differences in the occurrence of these. In Asia, CPS types Ia and III are the most common^17–19^ with a rare case of CPS type IX reported^20^. There are limited reports of *S. agalactiae* CPS typing from cases in Africa but those that have been typed were largely CPS type Ib^21^, with rare cases of CPS type IV in Northen Africa^22^, and more recently CPS type Ia in Ghana^23^. Although surveillance data is limited, CPS type Ib is most reported in North America and Central America^24^; however, CPS type Ia were recently described from Central America^25^. In the United States, most reported isolates are CPS type Ib^26^. In South America CPS type Ib is most common^26,27^; however, disease cases due to CPS type III isolates emerged in Brazil in 2016^28^ and rare cases of CPS type Ia have also been reported in Brazil^29^.

Based on MLST data, the majority of *S*. *agalactiae* isolates from tilapia group into several CCs. Most CPS type Ia isolates are CC7 (ST7)^24^, while most CPS type Ib isolates are in CC260 including several STs and non-typeable strains^24,29–31^, and CPS type III isolates belong to ST283 and ST491 of CC283^30^. There have been rare reports of CPS type Ia isolates belonging to ST103 of CC103^29^ and the CPS type IV isolates belong to ST2 (CC2)^22^.

In 2021 streptococcosis caused by CPS type Ia isolates emerged in Mexico^32,33^ and spread to other regions in Central and South America. Disease cases involving these emergent strains included all size classes of tilapia and presented different clinical signs than commonly observed with *S. agalactiae* CPS type Ib isolates; thus, such cases and the associated isolates were referred to as Ia-2021. A compounding factor in these epizootics was the failure of available vaccines which were formulated against CPS types Ia, Ib, and III to provide protection against the newly emerging strain.

The emergence of CPS type Ia-2021 devastated the tilapia industry in Latin America. Between 2021 and 2024, tilapia production in Mexico declined and caused farm closures^33^, and exportation of fresh fillets to the U.S. dropped from 13% to 1%^34^. In Honduras, the closure of a farming water body, and in Costa Rica, the shutdown of a production company, compounded the effects of the streptococcosis outbreak. These factors contributed to a decline in exports to the United States between 2022 and 2024 - 37% to 15% from Honduras and 18% to 11% from Costa Rica^34^. Colombia experienced a 26% reduction in exportation between 2023 and 2024 due to production issues related to streptococcosis^34^.

To address this emergent issue, the objectives of the present research were to: (1) document case reports of the disease, (2) reproduce clinical signs under laboratory conditions, (3) use comparative genomics to investigate the evolutionary history of the emergent strain, and (4) develop an assay to discriminate from other *S. agalactiae*.

## Results

### Outbreak investigation of Streptococcus agalactiae CPS type Ia-2021

#### Mexico

**Fig. 1:**
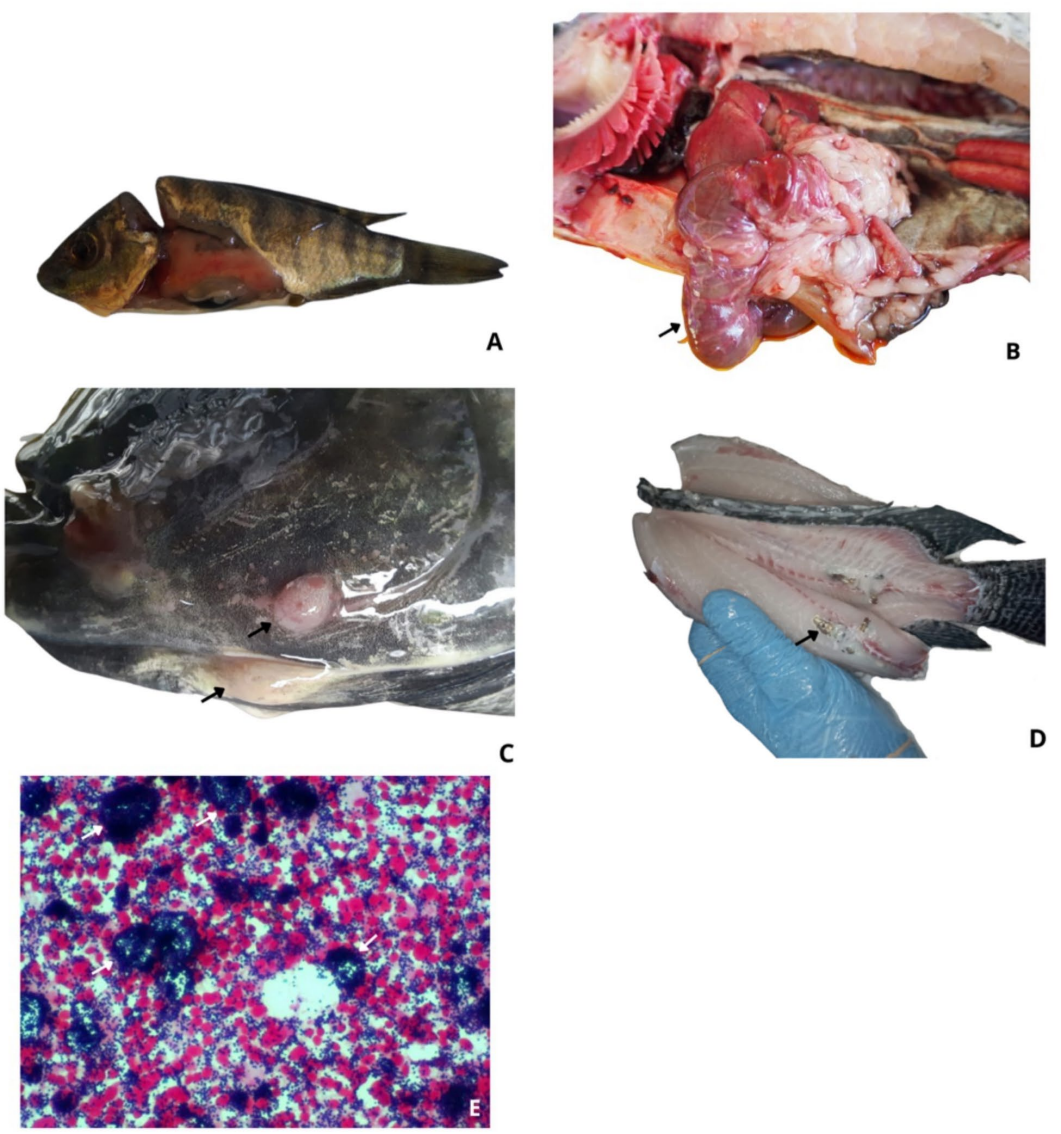
Gross lesions of streptococcosis observed in the field. (**A**) Nile tilapia alevin with hepatomegaly with hemorrhage, (**B**) Nile tilapia in grow-out phase with severe intussusception (arrow) and hemorrhage in testes, (**C**) pustules (arrows) in the surface of mandible, (**D**) severe and multifocal necrosis of muscle (arrow), and (**E**) Gram-stained spleen imprint (1000X) with abundant extracellular and intracytoplasmic Gram-positive cocci in macrophages (white arrows).

In September of 2021, alevins, grow-out, and broodstock tilapia vaccinated against *S*. *agalactiae* CPS types Ia and III were evaluated following unexpected high mortality events (30-40%). The water temperature during outbreaks was between 30 to 34°C. Alevins mainly exhibited hepatomegaly with hemorrhages (Fig. 1A), and grow-out and broodstock tilapia had pustules, some of them hemorrhagic. Gross lesions included pale liver, hemorrhaging of celomic organs, severe intussusception, and severe necrotic lesions in skeletal muscle (fillet) (Fig. 1B). Broodstock also had severe necrosis in gonads. Abundant Gram-positive cocci were observed in the liver and spleen of all fish by cytology and histopathology. Additionally, severe, diffuse necrotizing and granulomatous hepatitis, meningoencephalitis, meningomyelitis, discospondylitis, and myositis was observed associated with coccoid bacteremia. Beta hemolytic *Streptococcus agalactiae* CPS type Ia ST7 was recovered from all sampled fish, confirmed by PCR, and based upon the clinical signs of disease, isolates were considered CPS type Ia-2021.

#### Guatemala and El Salvador

In July of 2022, tilapia alevins and broodstock presented high mortality (20-25%). Gross lesions included corneal opacity, hepatomegaly, intestine with liquid content, intussusception, and severe necrosis of muscle (fillet). Histopathology revealed severe granulomatous meningitis, myositis, meningomeningitis, and discospondylitis with severe coccoid bacteremia. White beta hemolytic colonies were recovered and PCR confirmed *S*. *agalactiae* CPS type Ia in the pooled liver, spleen, and brain collected from alevins and broodstock and like the outbreaks in Mexico, isolates were considered CPS type Ia-2021.

#### Honduras

In August and September of 2022, an atypical mortality event in grow-out tilapia was reported (5-10%). Mortality in alevins and broodstock was milder but still higher than normal (increasing 0.5 to 1.0%). Severe hemorrhage in the celomic cavity, hepatomegaly, severe intussusception, meningoencephalitis, and epicarditis were observed in necropsy. Histopathology revealed severe coccoid bacteremia in the liver of alevins. Lymphocytic branchitis, gastritis, and granulomatous perihepatitis were diagnosed in grow-out fish. Beta hemolytic *Streptococcus agalactiae* CPS type Ia ST7 was isolated from all fish, confirmed by PCR, and as above, deemed the Ia-2021 strain based upon clinical signs. Isolate MO-Q-166 was sent to the USDA-ARS for further molecular characterization. Comorbidities with TiLV and edwardsiellosis were also detected.

#### Colombia

At the end of February and the first week of March of 2023, an atypical mortality event in alevins and grow-out tilapia was reported (> 25%). Mortality in broodstock was milder but higher than normal (increasing 0.5 to 2%). Gross lesions included pustules in the mandible, hemorrhagic lesions with exophthalmia, necrosis of the brain and hepatomegaly, severe intussusception, necrosis in the ovaries and testes, and necrotic lesions in skeletal muscle (fillet) (Fig. 1C, 1D). Cytology and histopathology revealed a severe bacteremia by Gram-positive coccoid bacteria in the liver, spleen, and kidney (Fig. 1E). Beta hemolytic isolates were confirmed as *S*. *agalactiae* CPS type Ia by PCR, deemed the CPS type Ia-2021 strain based upon clinical signs, and gDNA isolated from eight isolates was sent to the USDA-ARS for further molecular characterization.

#### Experimental challenge in Nile tilapia

**Fig. 2:**
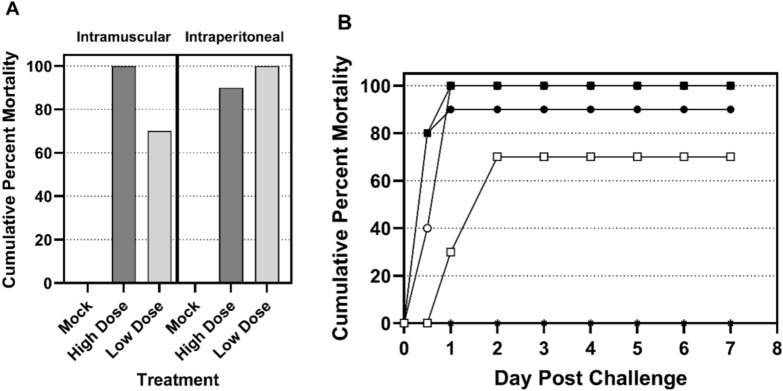
Cumulative percent mortality in Nile tilapia following challenge with the MO-Q-166 isolate of *Streptococcus agalactiae* CPS type Ia-2021. (**A**) Final cumulative percentage mortality following intramuscular and intraperitoneal injection with the high (6.9 × 10^5^ cfu fish^-1^) and low (6.9 × 10^2^ cfu fish^–1^) challenge doses. (**B**) Daily cumulative percent mortality following intraperitoneal injection with the high (●) or low (○) doses and intramuscular injection with the high (■) or low (□) doses.

Challenge of tilapia with the emergent *S. agalactiae* CPS type Ia-2021 isolate MO-Q-166 resulted in acute and high cumulative percent mortalities (CPM) using both injection routes. Final CPM following IP injection with the high and low doses resulted in 90 and 100% CPM, with all mortality occurring within 1 d post challenge (Fig. 2). The same doses delivered via IM injection resulted in CPM of 100 and 70%, respectively (Fig. 2A). At the low challenge dose, IM injection delayed mortality compared to IP injection in which no mortality occurred until 1 d post challenge and all mortalities occurred by 2 d post challenge (Fig. 2B). No mortality occurred in the mock challenged groups, and *S. agalactiae* was reisolated from 100% (9/9) of the challenge mortalities examined.

**Fig. 3:**
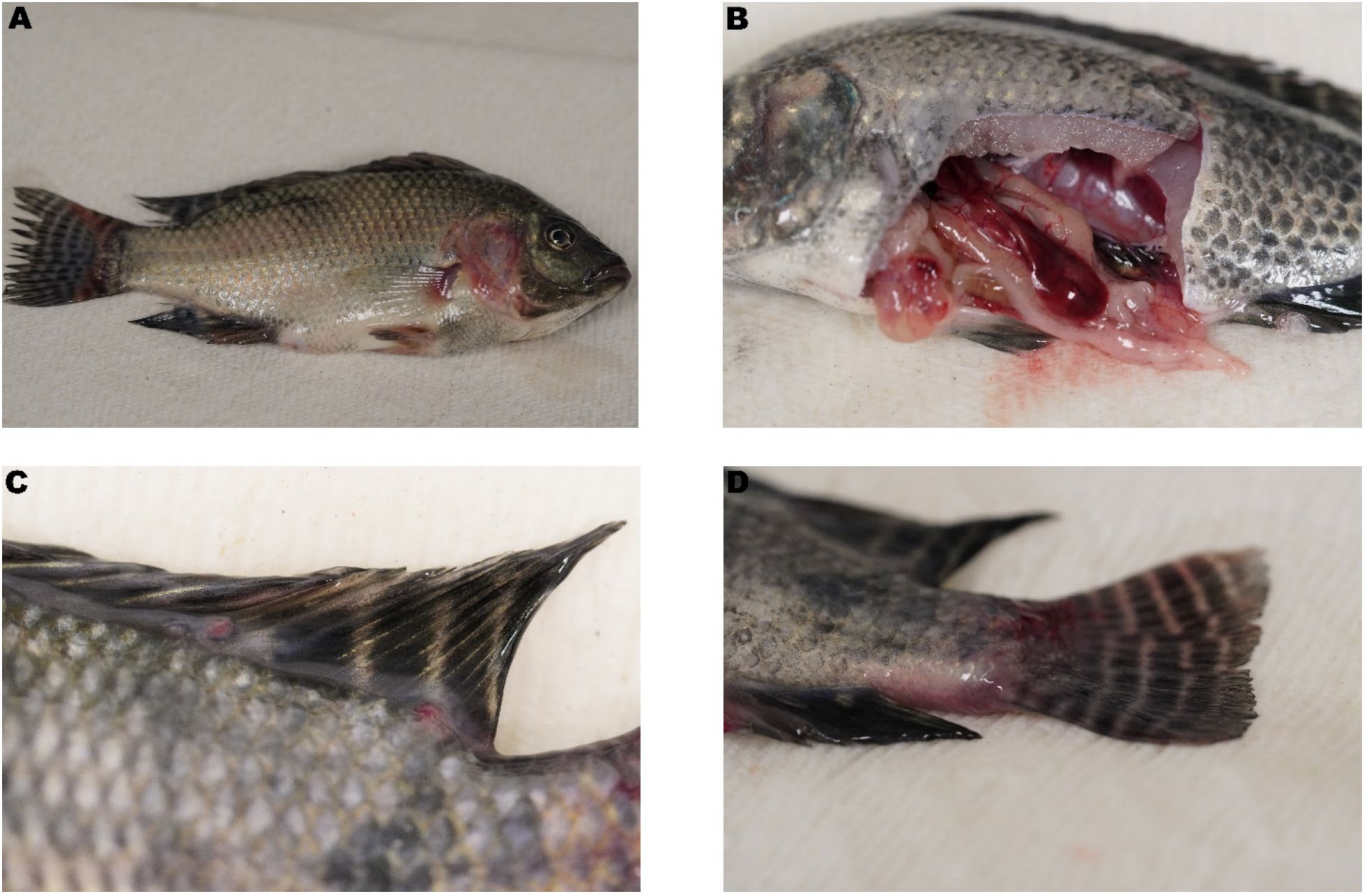
Gross lesions of streptococcosis in Nile tilapia following challenge with *Streptococcus agalactiae* CPS type Ia-2021 (MO-Q-166): (**A**) mortality following intraperitoneal injection showing hemorrhages of the gill operculum, fins, and skin; (**B**) mortality following intramuscular injection showing a pale and mottled liver, swollen kidney, and hemorrhaging and necrosis of the intestine; (**C**) survivor of challenge exhibiting pustules on the dorsal fin; (**D**) survivor of challenge exhibiting hemorrhagic and swollen caudal peduncle.

Clinical signs of disease were identical between the two routes of injection challenge. Moribund fish exhibited extreme erratic swimming behavior and long fecal strings in some cases. Additional external signs included hemorrhages of the gill operculum, fins, and in some cases the skin (Fig. 3A). Internally, moribund fish exhibited pale mottled livers, swollen kidneys and spleens, and hemorrhaging and necrosis of the intestine (Fig. 3B). Some surviving fish exhibited pustules on the dorsal fin (Fig. 3C) and swollen, hemorrhagic caudal peduncles (Fig. 3D).

#### Whole genome sequencing

High quality single contig, circularized genome assemblies with greater than 60X coverage were obtained following nanopore sequencing of the 12 Panel 1 isolates in-house and the 19 Panel 2 isolates by Plasmidsaurus (Supplemental Table.1). All genomes were 100% complete except for isolate M22063003 6.1K (99.91% complete) and contamination ranged from 0 to 0.89%. The genomes ranged in size from 2,064,658 to 2,267,872 bp and the GC (%) ranged from 35.6 to 35.9. The PGAP annotated genomes contained between 2,082 to 2,287 total predicted genes. Four CPS type Ia isolates harbored an ~ 4,441 bp plasmid with > 99.9% identity to an unnamed plasmid from *S. agalactiae* isolate CS2108 (NCBI accession no. CP123970.1). All isolates were assigned to ST7 and CC7 via MLST using the *S. agalactiae* PubMLST database. All genomes have been deposited at DDBJ/ENA/GenBank under the BioProject Number PRJNA1373282 and the accession numbers for each genome are provided in Table 1.

**Table 1:** Two panels of *Streptococcus agalactiae* isolates used for whole genome sequencing and comparative bacterial genomics.

Isolate name	Year	Country of origin	CPS type	NCBI accession
PANEL 1				
MO-Q-166	2022	Honduras	Ia-2021	JBTNNS000000000
CM-23-0180	2023	Colombia	Ia-2021	JBTNNR000000000
CM-23-0182	2023	Colombia	Ia-2021	JBTNNQ000000000
CM-23-0303	2023	Colombia	Ia-2021	JBTNNP000000000
CM-23-0366	2023	Colombia	Ia-2021	JBTNNO000000000
CM-23-0411	2023	Colombia	Ia-2021	JBTNNN000000000
CM-23-0473	2023	Colombia	Ia-2021	JBTNNM000000000
CM-23-0475	2023	Colombia	Ia-2021	JBTNNL000000000
CM-23-0735	2023	Colombia	Ia-2021	JBTNNK000000000
M23071204 1K	2023	Guatemala	Ia	JBTNNJ000000000
M23082505 14L	2023	Colombia	Ia-2021	JBTNNI000000000
M23082902 6K	2023	Mexico	Ia-2021	JBTNNH000000000
PANEL 2				
KU-MU-11Br	2001	Kuwait	Ia	JBTNNG000000000
FF18i2	2020	Haiti	Ia	JBTNNF000000000
M24011501 34.1B	2024	Colombia	Ia-2021	JBTNNE000000000
M23111304 12K	2023	Colombia	Ia-2021	JBTNND000000000
M23110601 3.1K	2023	Belize	Ia-2021	JBTNNC000000000
M23091802 6B	2023	Costa Rica	Ia	JBTNNB000000000
M23071302 9L	2023	Colombia	Ia-2021	JBTNNA000000000
M23061903 1.1K	2023	Honduras	Ia-2021	JBTNMZ000000000
M21110306 7	2021	Haiti	Ia	JBTNMY000000000
M23051104 40c	2023	Honduras	Ia-2021	JBTNMX000000000
M22122205 4.1K	2022	Honduras	Ia-2021	JBTNMW000000000
M22122204 7.1B	2022	Honduras	Ia-2021	JBTNMV000000000
M22090905 12B	2022	Costa Rica	Ia	JBTNMU000000000
M22063003 6.1K	2022	Mexico	Ia-2021	JBTNMT000000000
M21110306 19	2021	Haiti	Ia	JBTNMS000000000
M19092402 F8a	2019	Haiti	Ia	JBTNMR000000000
M24061702 9L	2024	Guatemala	Ia-2021	JBTNMQ000000000
M15112301 1	2015	Vietnam	Ia	JBTNMP000000000
M17081102 24	2017	Vietnam	Ia	JBTNMO000000000

The data provided for each isolate includes the isolate name, year of isolation, country of origin, designation as CPS type Ia-2021 or other Ia, and NCBI accession. All isolates were assigned to MLST sequence type 7/clonal complex 7 and originated from tilapia, except for KU-MU-11Br (mullet), M15112301 1 (barramundi), and M17081102 24 (barramundi).

#### Comparative bacterial genomics

**Fig. 4:**
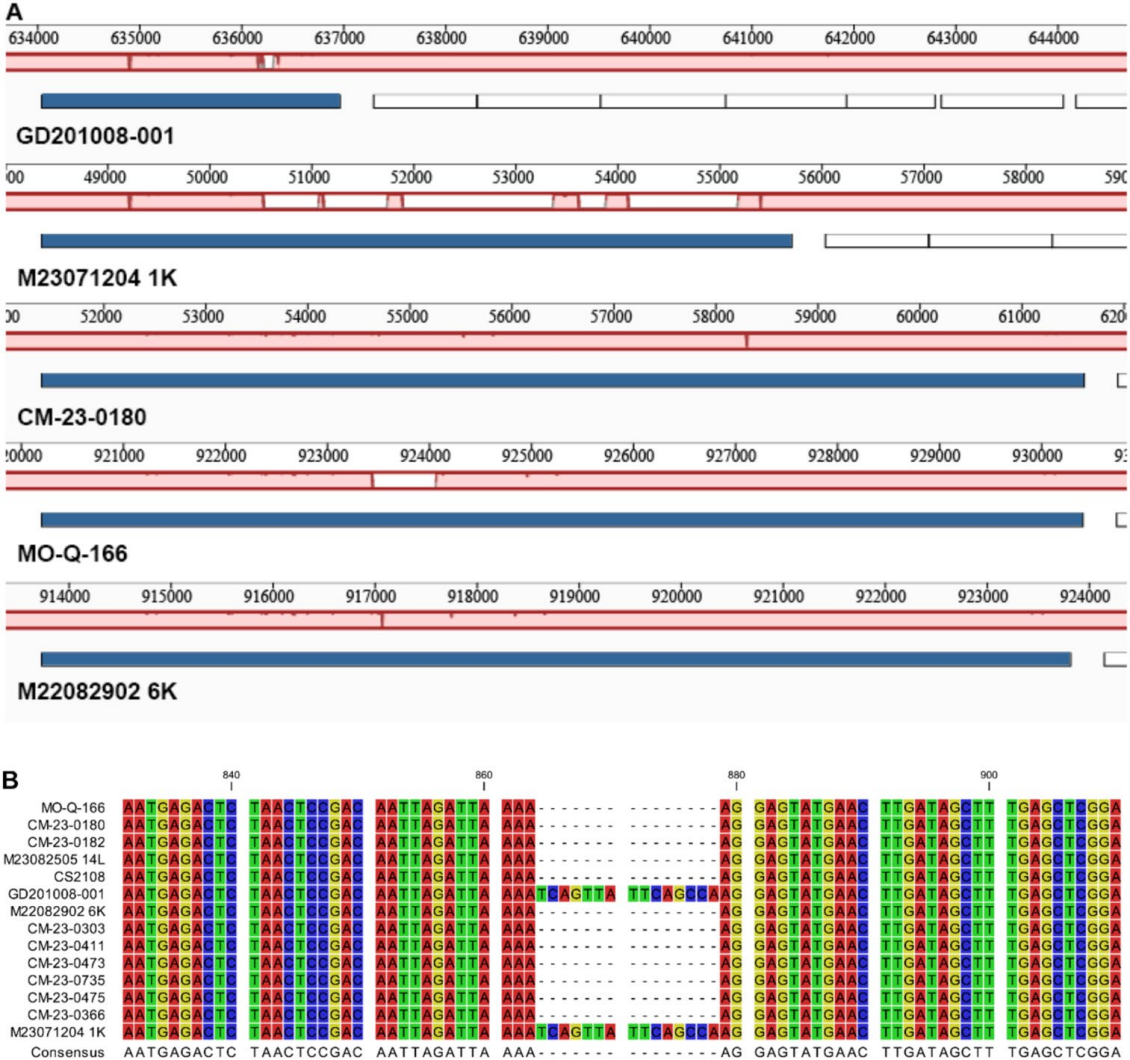
Analysis of the *srr-1* gene of *Streptococcus agalactiae*. (**A**) Mauve alignment of the *srr-*1 gene of the reference *Streptococcus agalactiae* CPS type Ia isolate GD201008-001 and the twelve Panel 1 isolates of which CPS type Ia M23071204 1K and CPS type Ia-2021 isolates CM-23-180, MO-Q-166, and M22082902 6K are shown. The full length *srr-*1 gene, ranging in size from 2,925 to 10,218 bp, is shown and colored in blue; (**B**) Multiple sequence alignment of the *srr-1* gene region containing the 15 bp deletion in *Streptococcus agalactiae* CPS type Ia-2021 isolates. The alignment includes the reference CPS type Ia isolate GD201008-001, CPS type Ia isolate M23071204 1K, and the CPS type Ia-2021 isolate CS2108 which was the only *S. agalactiae* isolate in the NCBI GenBank database containing the deletion.

Manual examination of the Muave alignment of the reference CPS type Ia genome GD201008-001 with the 12 *S. agalactiae* genomes (Panel 1) identified a striking difference in the length of the *srr-1* gene that encodes a serine rich repeat protein (Fig. 4A). The length of this gene in the reference genome was 2,925 bp, while the length of this gene in CPS type Ia-2021 isolates was about 3.5 times longer, or about 10,200 bp. The exception to this was the M23071204 1K (CPS type Ia) isolate, which carried an *srr-1* gene of 7,353 bp (Fig. 4A).

The *srr-1* gene was further examined to identify sequences that could be targeted by PCR to discriminate the reference CPS type Ia genome from the recent CPS type Ia-2021 isolates in which *srr-1* gene is much longer. Multiple sequence alignments revealed a 15 bp deletion in the conserved 5’ region of the *srr-1* gene at position 864 in the reference genome (Fig. 4B). This deletion was present in all CPS type Ia-2021 isolates in which the length of the gene was ~10,200 bp but absent in the CPS type Ia reference genome and the M23071204 1K isolate, which possessed an *srr-1* gene of intermediate length (7,353 bp). To determine if this deletion was unique to the emergent CPS type Ia-2021 isolates, a portion of the *srr-1* gene from isolate MO-Q-166 consisting of 500 bp up- and downstream of the deletion was queried against GenBank using BLASTn. The search returned a single sequence with 100% identity and containing an *srr-1* gene of 10,494 bp; all other publicly available isolates lacked the deletion. The matching genome (isolate CS2108; GenBank GCF_029917085.1) was deposited in 2023 and originated from a diseased tilapia in China.

#### Whole genome phylogeny

**Fig. 5:**
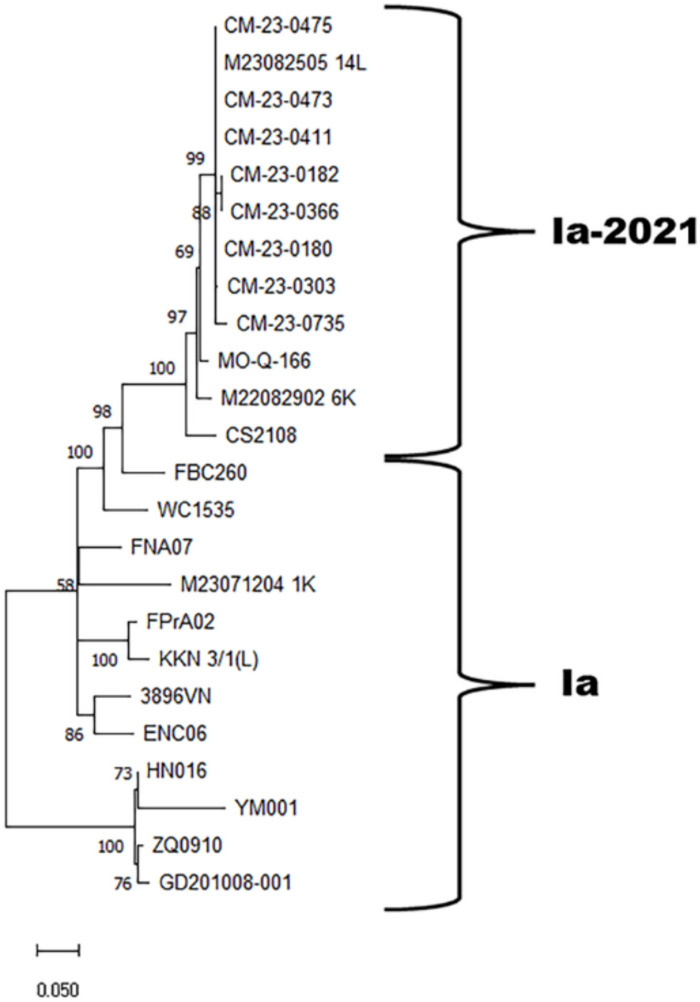
Genome based phylogenetic tree generated from the concatenated alignment of high-quality single nucleotide polymorphisms (SNPs) using CSI Phylogeny^35^. The evolutionary history was inferred by using the maximum likelihood method and Kimura 2-parameter model^36^. The percentage of trees in which the associated taxa clustered together is shown next to the branches. The tree is drawn to scale, with branch lengths measured in the number of substitutions per site. The analysis included 24 *Streptococcus agalactiae* CPS type Ia genomes and a total of 278 SNPs were included in the final dataset. GenBank accession numbers for isolates included in this analysis are provided in Table 1 and Supplemental Table 2.

To explore phylogenetic relationships among the CPS type Ia-2021 isolates with the 15 bp deletion and other Ia isolates lacking the deletion, a phylogenetic tree based on the concatenated alignment of high-quality single nucleotide polymorphisms (SNP) was generated. The analysis included 24 *S*. *agalactiae* CPS type Ia genomes, with a total of 278 SNPs included in the final dataset. The CPS type Ia-2021 isolates, including the CS2108 isolate from China (GenBank GCF_029917085.1), formed a strongly supported (bootstrap of 100) clade distinct from all other CPS type Ia isolates lacking the 15 bp deletion (Fig. 5). The remaining CPS type Ia isolates formed several clades with various levels of bootstrap support (Fig. 5).

#### Discriminatory PCR assay

**Fig. 6:**
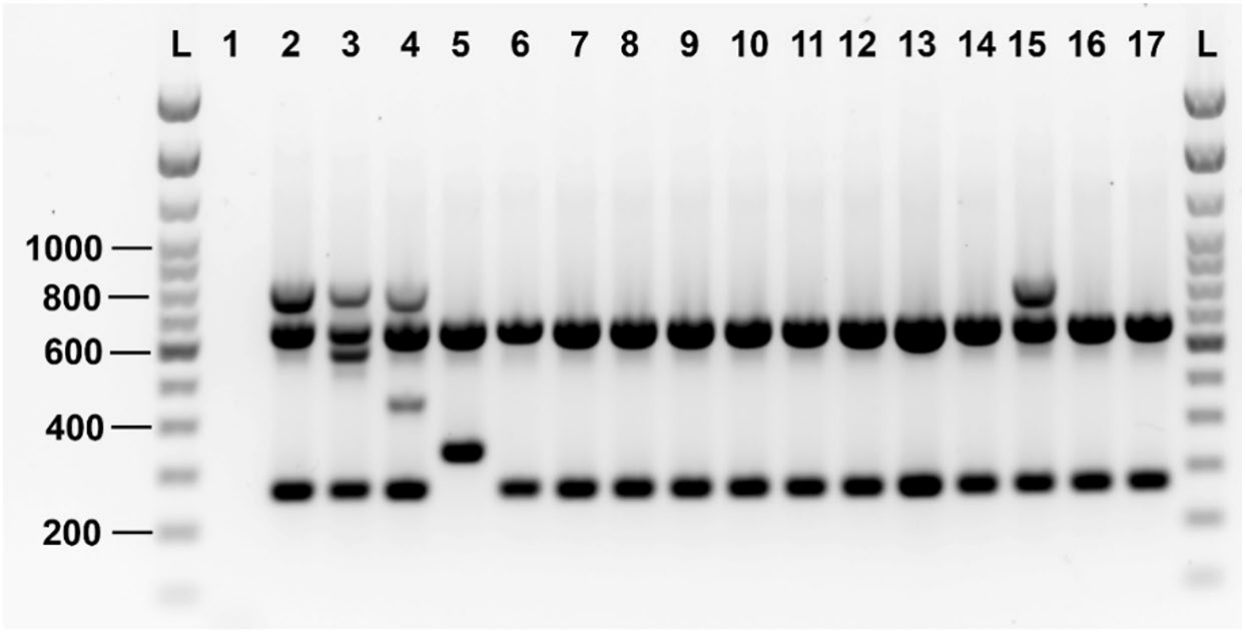
Agarose gel electrophoresis of PCR products amplified from gDNA of *Streptococcus agalactiae* isolates (Panel 1) using the new primer pair (Ia-old-F; Ia-old-R2) in conjunction with the multiplex PCR protocol of Imperi et al.^12^ as modified by Shoemaker et al.^26^. Lane L: 100 bp ladder; Lane 1: no template control; Lane 2: ATCC 12400 (CPS type Ia); Lane 3: ATCC 51487 (CPS type Ib); Lane 4: ATCC 13813 (CPS type II); Lane 5: ATCC 31475 (CPS type III); Lane 6: MO-Q-166; Lane 7: CM-23-0180; Lane 8: CM-23-0182; Lane 9: CM-23-0303; Lane 10: CM-23-0366; Lane 11: CM-23-0411; Lane 12: CM-23-0473; Lane 13: CM-23-0475; Lane 14: CM-23-0735; Lane 15: M23071204 1K; Lane 16: M23082505 14L; Lane 17: M23082902 6K; Lane L: 100 bp ladder.

Given the uniqueness of the 15 bp deletion in the *srr-1* gene of CPS type Ia-2021 isolates, the deletion was targeted for a discriminatory assay to differentiate between CPS type Ia-2021 and other Ia isolates. The forward primer was designed to overlap the deleted region so as not to bind to Ia-2021 isolates, while the reverse primer was designed to bind all isolates with the *srr-1* gene, yielding an 838 bp amplicon. The newly designed primers were tested in conjunction with the mPCR of Imperi et al.^12^ as modified by Shoemaker et al.^26^. All control CPS types Ia, Ib, II, and the M23071204 1K isolate yielded the expected 838 bp amplicon (Fig. 6), which was absent from the CPS type Ia-2021 isolates and the control CPS type III isolate. Thus, presence of the 838 bp amplicon, in addition to the expected 688 and 272 bp bands, indicates CPS type Ia isolates, while the absence of the 838 bp amplicon indicates CPS type Ia-2021.

#### Validation of assay specificity

**Fig. 7:**
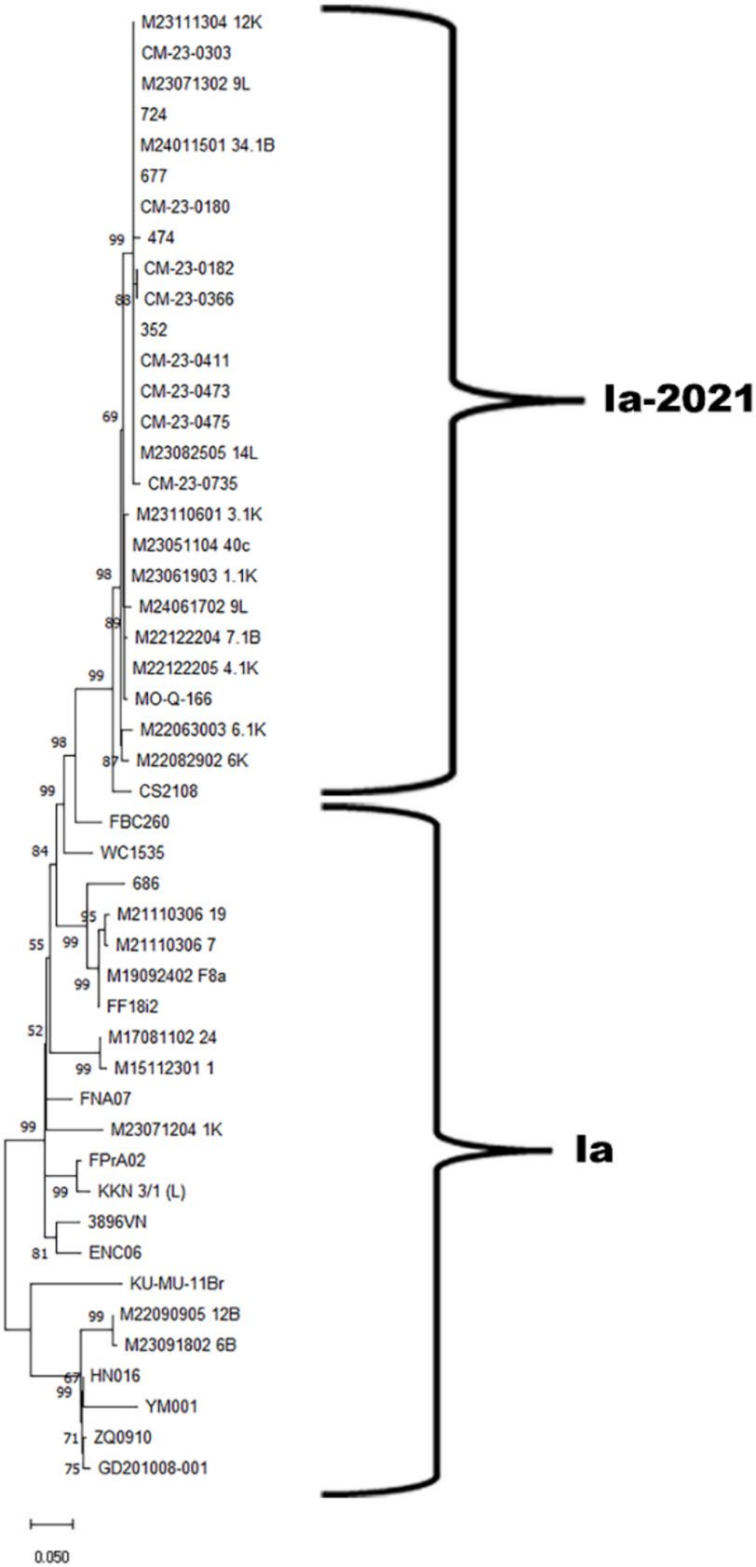
Genome based phylogenetic tree generated from the concatenated alignment of high-quality single nucleotide polymorphisms (SNPs) using CSI Phylogeny^35^. The evolutionary history was inferred by using the maximum likelihood method and Kimura 2-parameter model^36^. The percentage of trees in which the associated taxa clustered together is shown next to the branches. The tree is drawn to scale, with branch lengths measured in the number of substitutions per site. The analysis included 48 *Streptococcus agalactiae* CPS type Ia genomes and a total of 440 SNPs were included in the final dataset. GenBank accession numbers for isolates included in this analysis are provided in Table 1 and Supplemental Table 2.

**Fig. 8:**
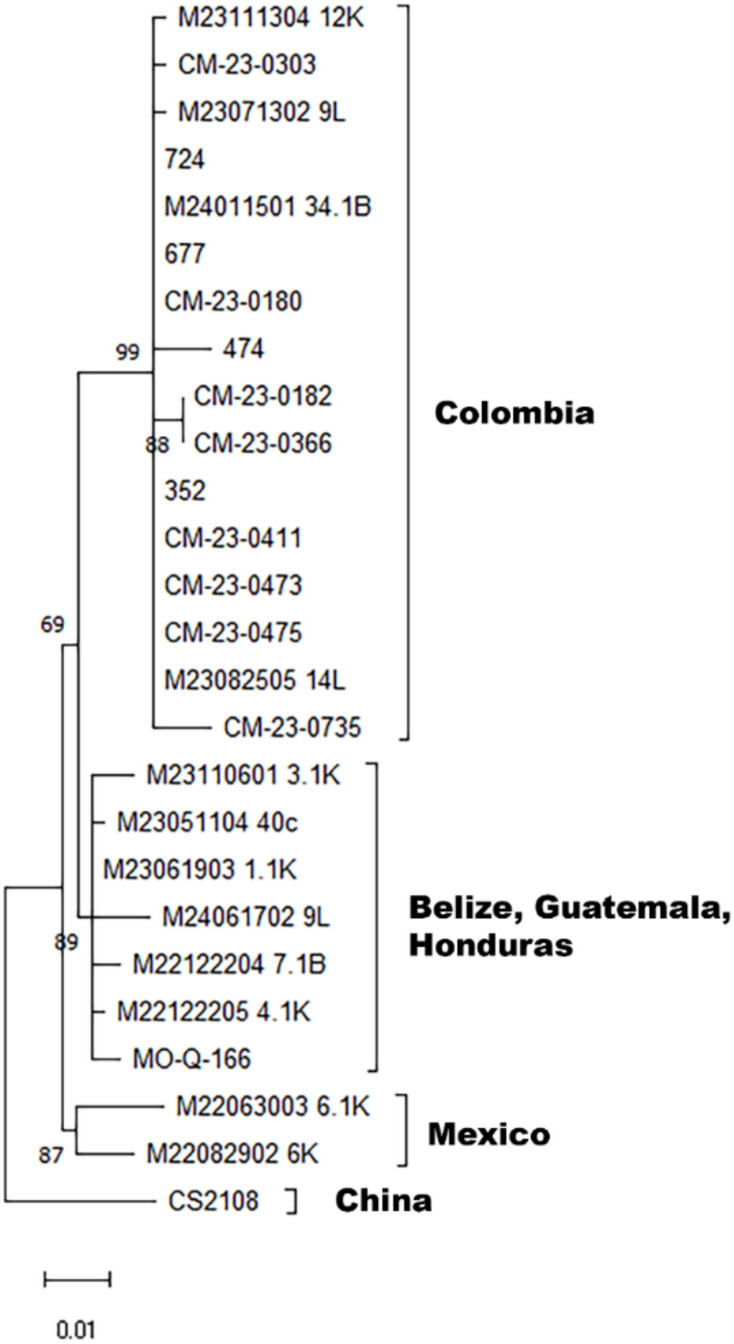
Subtree of the genome based phylogenetic tree generated from the concatenated alignment of high-quality single nucleotide polymorphisms displaying only the clade containing the *Streptococcus agalactiae* CPS type Ia-2021 isolates. Four subclades were formed with an apparent association with the geographic origin of the isolates. GenBank accession numbers for isolates included in this analysis are provided in Table 1 and Supplemental Table 2.

To validate the specificity of the assay, a second panel of nineteen *S. agalactiae* CPS type Ia isolates were blindly provided and typed using the new mPCR assay. Ten isolates were typed as the emergent CPS type Ia-2021 and nine isolates were typed as other Ia isolates (Supplemental Figure 1). Typing results were confirmed by whole genome sequencing and phylogenetic analysis. The analysis included 48 *S*. *agalactiae* CPS type Ia genomes, with a total of 440 SNPs included in the final dataset and generated a robustly supported tree delineating the emergent CPS type Ia-2021 isolates from other CPS type Ia isolates from tilapia (Fig. 7). The ten blind isolates that typed as Ia-2021 clustered with known Ia-2021 isolates, while the remaining nine blind isolates clustered with other CPS type Ia isolates, demonstrating the new assay is accurate and reliable in discriminating CPS type Ia isolates. Further, the clade containing the emergent CPS type Ia-2021 isolates contained four subclades with an apparent geographic association (Fig. 8). Three subclades contained only isolates from Colombia, Mexico, or the single isolate from China (CS2108). The fourth subclade was limited to isolates from Belize, Guatemala, and Honduras.

#### Optimization of mPCR assay for all S. agalactiae CPS types

**Fig. 9:**
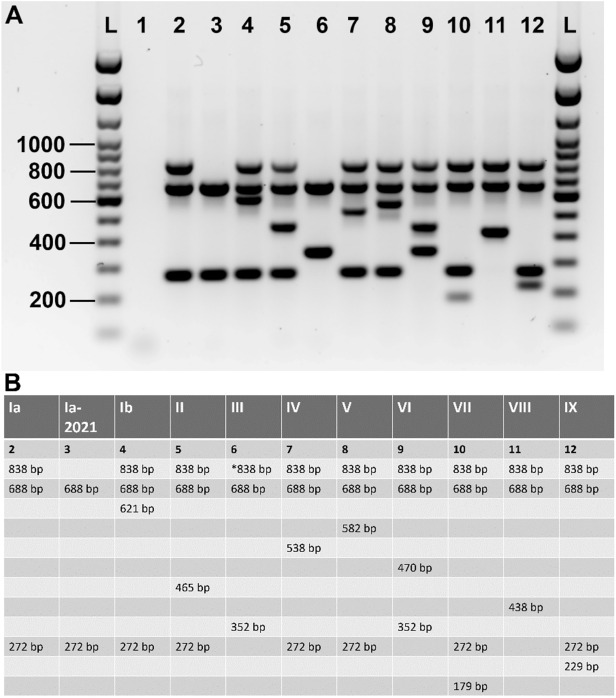
Optimized mPCR for CPS typing of *Streptococcus agalactiae*. (**A**) Agarose gel electrophoresis of PCR products amplified from gDNA of *Streptococcus agalactiae* isolates using the new primer pair (Ia-old-F; Ia-old-R2) in conjunction with the entire set of primers designed by Imperi et al.^12^. Lane L: 100 bp ladder; Lane 1: no template control; Lane 2: ATCC 12400 (CPS type Ia); Lane 3: M23082505 14L (Ia-2021); Lane 4: ATCC 51487 (CPS type Ib); Lane 5: ATCC 13813 (CPS type II); Lane 6: ATCC 31475 (CPS type III); Lane 7: BAA-2673 (CPS type IV); Lane 8: BAA-2672 (CPS type V); Lane 9: BAA-2671 (CPS type VI); Lane 10: BAA-2670 (CPS type VII); Lane 11: BAA-2669 (CPS type VIII); Lane 12: BAA-2668 (CPS type IX); (**B**) The expected number and size of amplicons for the ten CPS types and CPS type Ia-2021. * CPS type III isolates can harbor either a *srr-1* or *srr-2* gene and thus may be positive or negative for this amplicon.

The new primer pair for discriminating between CPS type Ia-2021 and other CPS type Ia isolates was included with the entire set of primers designed by Imperi et al.^12^ to provide a “one-stop shop” for *S. agalactiae* CPS typing capable of typing an unknown isolate to one of the ten CPS types and discriminating between Ia-2021 and other Ia isolates. Adjusting the annealing temperature to 57 °C and reducing the reaction volume to 15 µL were critical for the final protocol. Use of the optimized PCR protocol allowed for easy differentiation of the ten CPS types as well as discriminating between Ia-2021 and other Ia isolates (Fig. 9A). All control CPS types of *S. agalactiae* typed properly with the number and size of amplicons as described by Imperi et al.^12^. The addition of the new primer pair based upon the *srr-1* gene resulted in an 838 bp amplicon for all CPS types except for Ia-2021 (as expected) and the control CPS type III isolate. The number and size of amplicons for all ten CPS types and Ia-2021 are shown in Fig. 9B. Laboratory testing also confirmed the ability to use cells collected from sheep blood agar (SBA) plates as template (i.e., colony PCR) rather than extracted gDNA in the PCR assay with robust amplification needed for CPS typing (data not shown).

## Discussion

The emergence of CPS type Ia-2021 caused farm closures and substantial economic losses across multiple countries. Its ability to evade existing vaccines and spread rapidly has made it one of the most damaging bacterial threats currently facing tilapia aquaculture in the Americas. In the impacted countries, losses occurred in all phases of the production cycle, changing the epidemiological and clinical pattern of the disease. Epizootics of *S. agalactiae* typically occur during the grow-out phase of production when tilapia exceed 50 g, but are more frequent in larger fish, particularly those over 200 g up to market size^8^. The new presentation of the disease was acute and affected fingerlings from 0.5 g to grow-out and broodstock with severe hemorrhages in external and internal organs, intussusception, and life-threatening bacteremia (Fig. 1), and thus the reference to such cases and isolates as Ia-2021. Another compounding factor was vaccine failure in tilapia previously immunized against *S*. *agalactiae* CPS types Ia and III in Mexico, suggesting that the CPS type alone is not sufficient to induce immunity against *S. agalactiae* Ia-2021. The emergence of this strain highlighted the need for specific assays to distinguish its pathobiology, epidemiology, and identify CPS types associated with outbreaks to ensure effective vaccination.

Experimental laboratory challenge of tilapia with the CPS type Ia-2021 reproduced the clinical signs and lesions of disease observed in the industry and fulfilled Koch’s postulates. Clinical signs were identical following IP or IM injection and consisted of erratic swimming, hemorrhages of the operculum, fins, and skin, pale livers, and necrosis of the intestine (Fig. 3). These clinical signs are similar to those recently reported with streptococcosis cases caused by CPS type Ia in Mexico^33^. An acute disease pattern was observed using both routes of challenge with morbidity and mortality occurring by 12 h post challenge. Similarly, Avila-Castillo et al.^33^ also observed mortality in tilapia occurring by 12 h post-challenge by intraperitoneal injection with a recent CPS type Ia isolate from Mexico and mortality continued for 4 d. Intramuscular injection resulted in a slight delay in the onset of mortality (Fig. 2). Similar results were obtained by Soto et al.^37^ when different routes of challenge were tested using a CPS type Ib isolate of *S. agalactiae* in Nile tilapia. This research suggests CPS type Ia-2021 results in a more rapid disease progression than CPS type Ib in which mortality generally begins 3-4 days post challenge when administered by IM injection^37,38^. The use of IM injection may provide a reliable challenge model to recapitulate the disease for future laboratory research on CPS type Ia-2021.

Fish health experts in the region highlighted the need for a specific assay to discriminate this new strain from other CPS type Ia isolates. To address this need, genomic analysis of multiple emergent and regional *S. agalactiae* isolates was conducted to identify unique targets for a discriminatory PCR assay. One notable difference was the *srr-1* gene which was 3.5 times longer in the CPS type Ia-2021 isolates compared to the CPS type Ia reference genome (Fig. 4A).

The *srr-1* gene encodes a serine-rich repeat protein (SRRP), a large family of proteins unique to gram-positive bacteria with roles in host-pathogen interactions^39^. The features of SRRPs include a N-terminal signal peptide for transport, a short serine-rich repeat region, a binding region, a large serine-rich repeat region, and a C-terminal cell wall anchor domain^39^. The *srr-1* gene of *S. agalactiae* has been shown to bind to keratin 4^40^ and fibrinogen^41^, and gene deletion experiments have demonstrated a role in virulence^42,43^. Variation in the length of the portion of the gene encoding the large serine-rich repeat region has been previously noted for *S. agalactiae*^40^; however, the length of the *srr-1* gene and encoded protein of CPS type Ia-2021 isolates is unique among publicly available *S. agalactiae* genomes. It has been suggested the variable lengths of the larger serine-rich repeat region is thought to be necessary for extending the binding region of the protein away from the cell surface and capsule of the cell^44^. Further research is needed to fully understand the biological implications of a larger *srr-1* gene in CPS type Ia-2021 isolates and whether this may be involved in enhanced virulence in tilapia.

Further examination of the *srr-1* gene revealed the presence of a 15 bp deletion in the gene of CPS type Ia-2021 isolates (Fig. 4B). A BLASTn search revealed only one additional genome (CS2108 from China) carrying the same 15 bp deletion, and phylogenetic analysis showed all isolates with the deletion clustered together, supporting a shared evolutionary origin (Fig. 5). While it is not known whether there are any biological implications of this deletion for binding to host proteins and virulence, the results provided strong support for targeting the site of the deletion for a PCR assay to discriminate between CPS type Ia-2021 and other CPS type Ia isolates.

The discriminatory PCR assay reliably distinguished CPS type Ia-2021 isolates from other Ia strains based on absence of the 838 bp amplicon (Fig. 6). The *srr-1* gene is present in all ten CPS types except for some CPS type III isolates belonging to ST17 that contain a different SRRP gene, designated as *srr-2*^45,46^. This explains the presence of the 838 bp amplicon in the control CPS types Ib and II, and lack of the amplicon in the control CPS type III isolate ATCC 31475 (Fig. 6) which harbors the *srr-2* gene rather than the *srr-1* gene. Use of gDNA from CPS type III isolates with the *srr-1* gene resulted in amplification of the 838 bp amplicon (data not shown). Application of the modified mPCR to unknown CPS type Ia isolates was fully consistent with genome-based phylogeny, with Ia-2021 isolates clustering together and non-Ia-2021 isolates forming separate groups (Fig. 7), thereby validating the assay.

Phylogenetic analyses based upon the presence of SNPs in genomes have been shown to be useful for bacterial strain tracking and identifying associations among isolates of bacterial species with highly conserved genomes^47,48^. Kayansamruaj et al.^49^ performed a phylogenetic analysis of a small panel of *S. agalactiae* CPS type Ia (ST7/CC7) isolates using this method and the results clustered isolates according to host and geographic origin, providing a precedent for its use with *S. agalactiae*. Thus, this method was applied to the CPS type Ia (ST7/CC7) isolates from tilapia in this study. Examination of the clade containing the CPS type Ia-2021 isolates revealed four subclades. One subclade contained isolates from Belize, Guatemala, and Honduras. Two subclades only contained isolates from either Colombia or Mexico, while the fourth subclade only contained the single isolate from China (Fig. 8). Of potential interest was the finding that the subclades containing isolates from North, Central, and South America were more closely related to each other than the single isolate from China. It is unknown where the emergent strain originated from, but this data may suggest an independent emergence of the CPS type Ia-2021 in China or the isolate originated from China and then diverged in North, Central, and South America, or vice versa. Analysis of a larger number of isolates from China and other regions may provide information regarding the origin of CPS type Ia-2021 and will help determine whether geographic associations are valid or an artifact of limited sampling and small numbers of representative isolates from different regions. These results indicate SNP phylogenies of CPS type Ia-2021 isolates are useful for strain tracking which is important for understanding the spread and impact of this pathogen with implications for control and prevention.

The mPCR designed by Imperi et al.^12^ is a complex assay consisting of 19 primers for assigning unknown isolates to the ten CPS types. For simplification, some research has modified the assay to only include primers for the CPS types expected to be present^26,50^. For example, Shoemaker et al.^26^ modified the assay to only include the primers needed for identification of CPS types Ia, Ib, II, and III, and this modification was initially used in the present research with the new primers to discriminate between CPS type Ia-2021 and other Ia isolates (Fig. 6, Supplemental Fig. 1). However, eliminating primers for other CPS types may lead to incorrect CPS type assignment should rare or unexpected CPS types be encountered. For example, using the assay as initially performed in the present study, would assign CPS types IV, V, VII, and IX as CPS type Ia. This is because the primers necessary for amplicons needed to differentiate those CPS types from others would be missing and each of these CPS types would generate the 838, 688, and 272 bp amplicons expected for other CPS type Ia isolates (Fig. 9). Given this limitation, we optimized the assay to incorporate the two new primers for differentiating CPS type Ia-2021 and other Ia isolates with the full set of 19 primers designed by Imperi et al.^12^. This is important considering the reports of CPS types IV and IX causing disease and mortality in tilapia^20,22^ and thus the potential for improper typing. The optimized protocol allowed for robust amplification of all amplicons enabling easy interpretation of the number and size of amplicons to properly assign an unknown isolate to CPS type (Fig. 9). The optimized assay provides a “one-stop shop” for *S. agalactiae* CPS typing capable of assigning an unknown isolate to one of the ten CPS types and discriminating between CPS type Ia-2021 and other Ia isolates.

This research provides a comprehensive characterization of the emergent *S. agalactiae* CPS type Ia-2021 negatively impacting tilapia aquaculture in North, Central, and South America. The pathogen was found to be highly virulent utilizing injection challenge models and clinical signs of disease were similar to those observed in the field conditions. Whole genome sequencing and comparative genomics demonstrated the CPS type Ia-2021 isolates were phylogenetically distinct from other Ia isolates and led to the development of PCR assay to discriminate between these. The assay fills a critical need for epidemiological studies for surveillance of the emergent strain in global tilapia aquaculture and is an important tool for ensuring proper CPS types are incorporated into preventative vaccines. Additionally, this work provides a foundation for ongoing research to address vaccination strategies to combat streptococcosis, implement selective breeding for resistance to CPS type Ia-2021, understand pathology in tilapia infected with different CPS types, and identify differences between CPS type Ia-2021 and other Ia isolates which may lead to new prevention and control strategies.

## Methods

### Outbreak investigation of *Streptococcus agalactiae* CPS type Ia-2021 in Mexico, Guatemala, El Salvador, Honduras, and Colombia

Unusual initial mortality events in tilapia (alevins, grow-out, and broodstock) were observed in Mexico (September 2021; 30-40%), Guatemala (July 2022; > 20%), El Salvador (July 2022; 20-25%), Honduras (August 2022; 5-10%), and Colombia (February-March 2023; >25%). In each country (Mexico: 8 cases, 150 fish; Guatemala: 2 cases, 20 fish; El Salvador: 2 cases, 20 fish; Honduras: 8 cases, 180 fish; Colombia: 13 cases, 210 fish), necropsies were performed and samples were collected for cytology with Gram-stain and histopathology. In all countries, a pool of brain, liver, and spleen from each age group (alevins, grow-out, and broodstock) was used for bacterial isolation on blood agar. Isolates were tested by PCR for* S*. *agalactiae* and further characterized by CPS typing and whole genome sequencing. *Streptococcus agalactiae* isolates recovered from the atypical cases and assigned to CPS type Ia were considered the emergent strain and are referred to here as Ia-2021.

### Bacterial culture

*Streptococcus agalactiae* isolates were routinely cultured on SBA (Remel) at 28°C or in 50 mL centrifuge tubes containing 10-25 mL tryptic soy broth (TSB) at 28°C with shaking and cryopreserved in 20% glycerol at -80°C. For the experimental challenge, a single colony of CPS type Ia-2021 isolate MO-Q-166 was cultured in 25 mL TSB at 28°C with shaking at 175 rpm until an optical density (600 nm) of 0.8 was reached (~ 9 h). The culture was diluted 10^-2^ and 10^-5^ and these dilutions were used for high and low challenge doses. The undiluted inoculum was serially diluted in 10-fold dilutions from 10^-1^ to 10^-7^ and 50 µL volumes were spread plated onto SBA in duplicate. Colonies were counted after incubation of plates at 28°C for 48 h and the number of colony-forming units (cfu) mL^-1^ were determined using standard procedures. For whole genome sequencing, *S. agalactiae* isolates were cultured from single colonies in 10 mL TSB for ~17 h at 28°C with shaking. Cells were pelleted by centrifugation and genomic DNA was extracted using the gram-positive protocol of the Qiagen DNeasy Blood and Tissue Kit (Qiagen). The concentration and quality of DNA was determined using a NanoDrop One^C^ (ThermoFisher Scientific) targeting a 260/280 ratio of 1.7-2.0 and 260/230 ratio of 1.8-2.2.

### Rearing fish for experimental challenge

Nile tilapia were obtained from stocks reared indoors for research at the U.S. Department of Agriculture (USDA) Agricultural Research Service (ARS) Aquatic Animal Health Research Unit (AAHRU) in Auburn, Alabama, USA. Prior to experimentation, the fish were determined to be negative for *S. agalactiae* by plating brain tissue from a subsample of 10 fish onto SBA, incubating for 4 d at 28°C, and examining for bacterial growth. Prior to bacterial challenge, fish were maintained in a 378 L tank supplied with a blended mix of flow-through dechlorinated municipal water and well water at approximately 28°C with supplemental aeration provided by air stones. Fish were fed a commercial floating pelleted feed (Skretting USA) at 2% body weight per day. Fish were not fed the day of or before challenge. Following bacterial challenge, fish were placed into 60 L tanks (51 L water volume) supplied with flow-through water at a rate of 0.5 L min^-1^ and supplemental aeration was provided with air stones. Water temperature (27.4 ± 0.2°C) and dissolved oxygen (7.6 ± 0.2 ppm) were recorded daily throughout the challenge. All fish handling procedures were approved by the Institutional Animal Care and Use Committee at the USDA-ARS AAHRU, conformed to USDA-ARS Policies and Procedures 130.4.v5, and according to the ARRIVE guidelines^51^.

### Experimental challenge in Nile tilapia

For experimental challenge with the CPS type Ia-2021 isolate MO-Q-166, groups of 10 tilapia (average weight of 32.5 g) were challenged with (1) high dose using intraperitoneal (IP) injection, (2) low dose using IP injection, (3) high dose using intramuscular (IM) injection, or (4) low dose using IM injection. Additionally, groups of 10 tilapia were mock challenged by IP or IM injection with sterile TSB, and treatments were randomly assigned to tanks. Prior to challenge, fish were anesthetized in water containing 120 mg L^–1^ Syncaine (Syndel) buffered with sodium bicarbonate (1:1) and injected either IP or IM with 100 µL suspensions containing the high dose of 6.9 × 10^5^ cfu or the low dose of 6.9 × 10^2^ cfu with 25- gauge needles. Following injection, fish were placed into their respective tanks, allowed to recover from anesthesia and observed for morbidity and mortality at least twice daily for 7 d. Dead and terminally moribund fish (euthanized with an overdose of Syncaine buffered 1:1 with sodium bicarbonate) were removed and recorded. Fish were examined for clinical signs of streptococcosis and reisolation of *S. agalactiae* was attempted on at least 20% of the daily mortalities from each tank by culturing brain tissue onto SBA. Reisolation was considered positive if colonies phenotypically consistent with *S. agalactiae* CPS type Ia-2021 were visualized on plates after incubation for 48 h at 28°C.

### Collection of isolates

Two panels of *S. agalactiae* CPS type Ia isolates were collected and analyzed (Table 1). The first panel of isolates contained nine isolates originating from streptococcosis cases in tilapia with clinical signs consistent with the emergent CPS type Ia-2021 (MO-Q-166, CM-23-0180, CM-23-0182, CM-23-0303, CM-23-0366, CM-23-0411, CM-23-0473, CM-23-0475, and CM-23-0735) and three other isolates from recent streptococcosis cases in the region. The second panel of nineteen isolates contained recent tilapia isolates from streptococcosis cases in North, Central and South America and contained archival isolates collected prior to 2021. Only three isolates originated from species other than tilapia, and these included KU-MU-11Br which was isolated from mullet *Planiliza klunzingeri* in Kuwait^52^, and isolates M15112301 1 and M17081102 24 from barramundi *Lates calcarifer* in Vietnam. The CPS type of each *S. agalactiae* isolate was determined using the multiplex PCR (mPCR) assay of Imperi et al.^12^ as modified by Shoemaker et al.^26^.

### Genome sequencing

The genomes from the first panel of 12 *S. agalactiae* isolates were sequenced in-house at the AAHRU. The Rapid Sequencing Kit v14 with the Rapid Barcoding Kit 24 v14 (Oxford Nanopore Technologies; ONT) was used to create the libraries and sequenced on an GridION (ONT) using a R10.4.1 flow cells (ONT), which have been shown to produce near-perfect bacterial genomes without short read or reference polishing^53,54^. Basecalling was performed on the resultant POD5 files in the Super Accurate (SUP) configuration using Dorado basecaller within the GridION to produce FASTQ files. FASTQ files were used to assemble the genomes using Flye v2.9.2-b1786 with the options --nano-hq, --asm-coverage 50, and -g 2m. All other parameters were default.

Whole genome sequencing of the second panel of 19 isolates was performed by Plasmidsaurus (Louisville, KY, USA) using gDNA extracted as described above and Oxford Nanopore Technology. An amplification-free long-read sequencing library was created using v14 library prep chemistry including minimal fragmentation of the input genomic DNA in a sequence-independent manner. The library was sequenced with a primer-free protocol using R10.4.1 flow cells (ONT). High quality genome assemblies were produced by removing the bottom 5% worst fastq reads via Filtlong v0.2.1 with default parameters. The reads were downsampled to 250 Mb via Fitlong to create a rough sketch of the assembly with Miniasm v0.3. Information from the Miniasm assembly was used to re-downsample the reads and then assembled with Fly v2.9.1 and polished via Medaka v1.8.0. The assembled genomes were annotated by RAST^55–57^ and submitted to NCBI for annotation using the Prokaryotic Genome Annotation Pipeline (PGAP, version 6.10). The quality of each genome assembly was assessed for completeness and contamination using CheckM (v1.2.2). The ST and CC of each isolate was determined using the MLST scheme developed for *S. agalactiae*^15^. FASTA files of the whole genome sequences were queried using the *S. agalactiae* typing database at PubMLST^16^.

### Comparative bacterial genomics

Comparative genomics was performed to identify potential unique elements within the emergent *S. agalactiae* CPS type Ia-2021 genomes and identify regions that could be targeted to develop a discriminatory assay to differentiate the CPS type Ia-2021 isolates from other CPS type Ia isolates. Initial analysis compared 12 genomes (Panel 1) to a reference CPS type Ia genome (isolate GD201008-001; NCBI RefSeq GCF_000299135.1). Annotated genomes were imported into Geneious Prime v.2024.0.5 and aligned using the progressiveMauve algorithm and default parameters^58^. Resultant alignments were manually examined to identify unique genes and/or regions.

### Whole genome phylogeny

To explore phylogenetic relationships among CPS type Ia-2021 and other Ia isolates, a phylogenetic tree based on the concatenated alignment of high-quality single nucleotide polymorphisms (SNP) was generated using CSI Phylogeny^35^ and the default parameters. The genome of *S. agalactiae* CPS type Ia isolate GD201008-001 was used as the reference and the analysis included the 12 genome sequences from Panel 1 (Table 1) and 11 other publicly available *S. agalactiae* CPS type Ia genomes from tilapia (Supplemental Table 2). The FASTA alignment of the concatenated high-quality SNP’s was downloaded and imported into MEGA11^59^ and the evolutionary relatedness was inferred using maximum likelihood method and the Kimura 2-parameter model^36^. Initial trees for the heuristic search were obtained automatically by applying Neighbor-Join and BioNJ algorithms to a matrix of pairwise distances estimated using the Maximum Composite Likelihood approach and then selecting the topology with superior log likelihood value.

### Development of primers/discriminatory PCR assay

Following the identification of a region to target for a discriminatory assay, the gene sequence from CPS type Ia-2021 isolate MO-Q-166 was searched against the core nucleotide database at NCBI using BLASTn to determine if the genomic feature identified was common among other *S. agalactiae* genomes or unique to CPS type Ia-2021 isolates. Then, the sequences of the gene were retrieved from the genomes of the 12 Panel 1 isolates and the reference genome GD201008-001 and then subjected to a multiple sequence alignment using CLC Genomics Workbench (version 24.0.1, Qiagen). A forward primer (Ia-old-F) was designed to specifically bind to a unique DNA sequence present only in other CPS type Ia isolates (non-Ia-2021) and a reverse primer (Ia-old-R2) was designed to specifically bind to any isolate with the target sequence (Supplemental Table 3). Primers were designed to be integrated with the mPCR for the CPS typing of *S. agalactiae*^12^. The primer design function of CLC Genomics Workbench was used to identify primers with the following constraints: melting temperatures compatible with annealing temperatures (54 and 56°C) used by Imperi et al.^12^ and resulting in an amplicon size greater than any amplicons generated in the mPCR (> 688 bp).

Initially, the new primer set was tested with the mPCR of Imperi et al.^12^ as modified by Shoemaker et al.^26^ to only use the primers specific for CPS types Ia, Ib, II, and III (primers 3-11; Supplemental Table 3). PCR was performed with HotStarTaq *Plus* Master Mix Kit (Qiagen), and the optimized concentrations of each component in the 25 μL reaction mixture were as follows: 12.5 μL of 2× HotStarTaq *Plus* master mix, 2 μL of primer mix (final concentration of 0.18 μM for each primer: cpsL-F, cpsL-R, cpsG-F, CpsG-R, CpsG-2-3-6-R, cpsJ-2-4-F, cpsJ-2-R, cpsJ-Ib-F, and cpsJ-Ib-R), 0.5 µL of 10 µM Ia-old-F (final concentration, 0.2 µm), 0.5 µL of 10 µm Ia-old-R2 (final concentration, 0.2 µM), 2.5 μL of 10× CoralLoad concentrate, 25 ng template gDNA, and nuclease free water to volume. Positive controls consisted of 20 ng gDNA from CPS types Ia (ATCC 12400), Ib (ATCC 51487), II (ATCC 13813), and III (ATCC 31475), and no template negative controls were included. PCR amplification was performed with a Mastercycler nexus gradient thermal cycler (Eppendorf) using the following cycling protocol: one cycle at 95°C for 5 min; 15 cycles of 60 s at 94°C, 60 s at 54°C and 120 s at 72°C; 25 cycles of 60 s at 94°C, 60 s at 56°C and 120 s at 72°C; and a final cycle of 10 min at 72°C^12^. Following amplification, 5 μL of each PCR product was run on a 1.5% (w/v) agarose gel in Tris–acetate–EDTA (TAE buffer). Gels were precast with 1× SYBR Safe DNA gel stain (Invitrogen), and the products along with a concurrently run molecular weight standard (Invitrogen) were visualized using ultraviolet transillumination.

### Validation of assay specificity

To validate the newly developed primers for their specificity to differentiate CPS type Ia and Ia-2021 isolates, a second panel (Panel 2) of 19 *S. agalactiae* CPS type Ia isolates (Table 1) were blindly provided, typed using the mPCR assay, and assigned to either Ia or Ia-2021. Subsequently, the whole genomes of these isolates were sequenced by Plasmidsaurus and used to generate a phylogenetic tree based on the concatenated alignment of high-quality SNPs as above. This analysis consisted of using the genome of *S. agalactiae* CPS type Ia isolate GD201008-001 as the reference and included the 19 Panel 2 isolates, the 12 Panel 1 isolates, and 11 publicly available CPS type Ia genomes from tilapia that were used in the initial phylogenetic analysis above (Table 1; Supplemental Table 2). Additionally, five new genomes from CPS type Ia isolates obtained from streptococcosis cases in Colombia (ICA and Agrosavia, unpublished) were included (Supplemental Table 2). The FASTA alignment of the concatenated high-quality SNPs was downloaded, imported into MEGA11, and evolutionary relatedness of the genomes was inferred as above.

### Optimization of mPCR assay for all S. agalactiae CPS types

The mPCR was optimized for the ability to clearly assign any *S. agalactiae* isolate to CPS type using the full set of primers developed by Imperi et al.^12^ and the new primers developed in the present study (n = 21; Supplemental Table 3). To do this, numerous optimization strategies were employed including use of different Taq polymerase kits, primer concentrations, annealing temperatures, reaction volumes, cycle numbers, template DNA concentrations, and use of additives such as Q-solution (Qiagen). The optimal protocol used AllTaq Master Mix Kit (Qiagen) and the following PCR conditions. All primers were ordered from Integrated DNA Technologies (IDT) lab ready at 100 µM and a pool consisting of all primers was prepared by combining 10 µL of all primers except CpsI-Ia-6-7-F and CpsI-7-9-F, in which 16 µL were used with 178 uL of nuclease free water. This provided final concentration of 2.5 µM for all primers, except for CpsI-Ia-6-7-F and CpsI-7-9-F in which the final concentrations were 4 µM. Optimized reaction mixtures (15 μL) consisted of 5 μL of 4× AllTaq master mix, 2 μL of the primer pool, 0.16 μL of 125× master mix tracer, 25 ng of template gDNA, and nuclease free water to volume. Positive controls consisted of gDNA from CPS types Ia (ATCC 12400), Ia-2021 (M23082505 14 L), Ib (ATCC 51487), II (ATCC 13813), III (ATCC 31475), IV (BAA-2673), V (BAA-2672), VI (BAA-2671), VII (BAA-2670), VIII (BAA-2669), IX (BAA-2668), and no template negative controls were included. PCR amplification was performed with a Mastercycler nexus gradient thermal cycler (Eppendorf) using the following cycling protocol: one cycle at 95°C for 5 min; 40 cycles of 60 s at 94°C, 60 s at 57°C and 120 s at 72°C; and a final extension of 10 min at 72°C. Following amplification, PCR products were detected by subjecting 1 μL of the PCR to 1.5% (w/v) agarose gel electrophoresis in TAE buffer. Gels were precast with 1× SYBR Safe DNA gel stain (Invitrogen), and the products along with a concurrently run molecular weight standard (Invitrogen) were visualized using ultraviolet transillumination. Optimal conditions were defined as those that produced consistent amplification of all target amplicons at their expected sizes, minimal nonspecific products, balanced band intensities across targets, high specificity against non-target CPS types, and reproducible results across replicate reactions and DNA concentrations.

## Data availability 

All genomes have been deposited at DDBJ/ENA/GenBank under the BioProject Number PRJNA1373282 and the accession numbers for each genome are provided in Table 1. Other data generated during and/or analysed during the current study are available from the corresponding author on reasonable request.

## Supplementary Information

Supplementary Information.
