# Supplementary material for: Emergence, identification, and characterization of a novel Streptococcus agalactiae CPS type Ia ST7 strain (Ia-2021) causing large scale mortalities in tilapia aquaculture
Source: Sci Rep. 2026 May 9;16:20338. doi: 10.1038/s41598-026-52208-0 (PMC13328444; doi:10.1038/s41598-026-52208-0)
Supplement: Supplementary file 1 — Supplementary Information. [file 41598_2026_52208_MOESM1_ESM.docx]

Supplementary information

**Emergence, identification, and characterization of a novel *Streptococcus agalactiae* CPS type Ia ST7 strain (Ia-2021) causing large scale mortalities in tilapia aquaculture**

Benjamin R. LaFrentz^1^*, Paola Barato^2^, William R. Keleher^3^, Amber E. Johnston^1,4^, Megan Raftery, Jason W. Abernathy^1^

^1^ United States Department of Agriculture – Agricultural Research Service (USDA-ARS), Aquatic Animal Health Research Unit, Auburn, Alabama 36832, USA

^2^ CORPAVET and MolecularVet SAS, Calle 24 # 3 – 27, Neiva, Huila, Colombia, and MolecularVet US LLC, Davie, Florida 33330, USA

^3^ Kennebec River Biosciences, Richmond, ME 04357, USA

^4^ Present address: Aquaculture Research Institute, University of Maine, Orono, ME 04469, USA

*** Corresponding Author:** Benjamin R. LaFrentz; Email: [benjamin.lafrentz@usda.gov](mailto:benjamin.lafrentz@usda.gov)

**
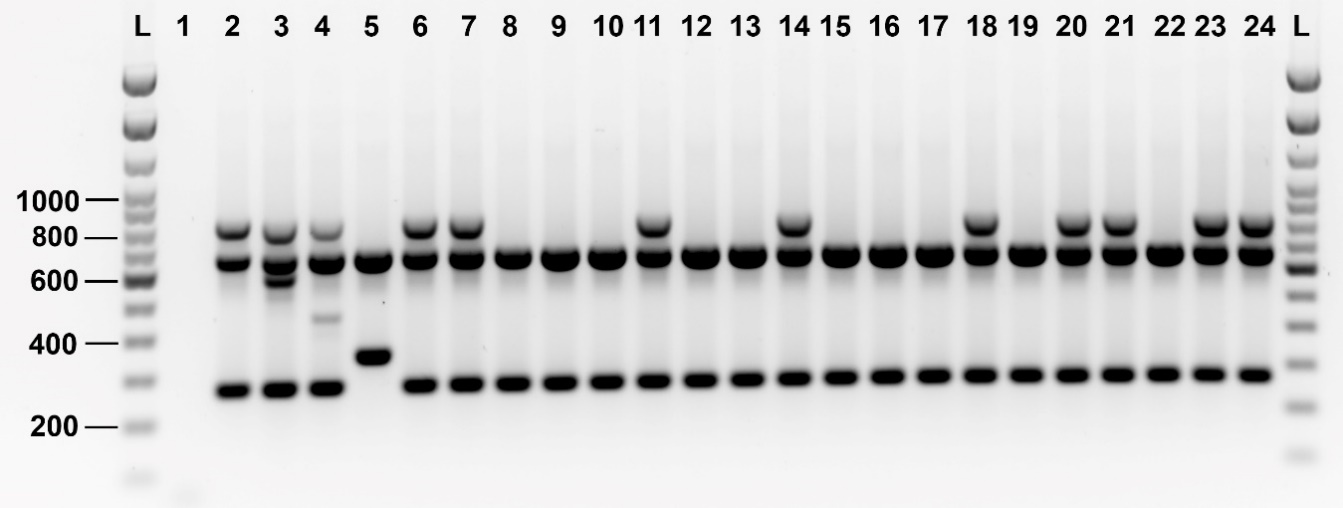
**

**Supplemental Figure 1.** Agarose gel electrophoresis of PCR products amplified from gDNA of *Streptococcus agalactiae* isolates (Panel 2) using the new primer pair (Ia-old-F; Ia-old-R2) in conjunction with the multiplex PCR protocol of Imperi et al. ^1^ as modified by Shoemaker et al. ^2^. Lane L: 100 bp ladder; Lane 1: no template control; Lane 2: ATCC 12400 (CPS type Ia); Lane 3: ATCC 51487 (CPS type Ib); Lane 4: ATCC 13813 (CPS type II); Lane 5: ATCC 31475 (CPS type III); Lane 6: KU-MU-11Br; Lane 7: FF18i2; Lane 8: M24011501 34.1B; Lane 9: M23111304 12K; Lane 10: M23110601 3.1K; Lane 11: M23091802 6B; Lane 12: M23071302 9L; Lane 13: M23061903 1.1K; Lane 14: M21110306 7; Lane 15: M23051104 40c; Lane 16: M22122205 4.1K; Lane 17: M22122204 7.1B; Lane 18: M22090905 12B; Lane 19: M22063003 6.1K; Lane 20: M21110306 19; Lane 21: M19092402 F8a; Lane 22: M24061702 9L; Lane 23: M15112301 1; Lane 24: M17081102 24; Lane L: 100 bp ladder.

**Supplemental Table 1.** Summary of statistics for the whole genome sequencing, assembly and annotation of the 31 *Streptococcus agalactiae* isolates. The data provided includes the isolate name, genome coverage, N_50_ read length, genome size, GC content, total number of predicted genes and number of plasmids identified.

| **Isolate Name** | **Genome Coverage** | **Read N_50_ (bp)** | **Genome Size (bp)** | **GC content (%)** | **Total No. of predicted genes** | **No. of plasmids** |
| --- | --- | --- | --- | --- | --- | --- |
| **PANEL 1** |  |  |  |  |  |  |
| MO-Q-166 | 108× | 7,877 | 2,113,329 | 35.7 | 2,133 | 0 |
| CM-23-0180 | 180× | 13,821 | 2,114,571 | 35.7 | 2,135 | 0 |
| CM-23-0182 | 331× | 15,085 | 2,114,451 | 35.7 | 2,134 | 0 |
| CM-23-0303 | 80× | 14,615 | 2,115,706 | 35.7 | 2,135 | 0 |
| CM-23-0366 | 76× | 11,245 | 2,114,608 | 35.7 | 2,135 | 0 |
| CM-23-0411 | 74× | 15,085 | 2,114,605 | 35.7 | 2,134 | 0 |
| CM-23-0473 | 77× | 16,184 | 2,114,568 | 35.7 | 2,134 | 0 |
| CM-23-0475 | 78× | 12,753 | 2,114,569 | 35.7 | 2,134 | 0 |
| CM-23-0735 | 76× | 10,618 | 2,114,574 | 35.7 | 2,134 | 0 |
| M23071204 1K | 60× | 7,886 | 2,186,441 | 35.8 | 2,214 | 1 |
| M23082505 14L | 374× | 11,152 | 2,114,605 | 35.7 | 2,135 | 0 |
| M23082902 6K | 70× | 10,348 | 2,117,258 | 35.7 | 2,137 | 0 |
| **PANEL 2** |  |  |  |  |  |  |
| KU-MU-11Br | 98× | 7,916 | 2,105,573 | 35.7 | 2,126 | 0 |
| FF18i2 | 64× | 7,759 | 2,120,609 | 35.8 | 2,141 | 1 |
| M24011501 34.1B | 102× | 5,740 | 2,114,611 | 35.7 | 2,132 | 0 |
| M23111304 12K | 101× | 11,109 | 2,114,611 | 35.7 | 2,134 | 0 |
| M23110601 3.1K | 100× | 10,651 | 2,112,520 | 35.7 | 2,134 | 0 |
| M23091802 6B | 101× | 10,158 | 2,064,591 | 35.7 | 2,082 | 0 |
| M23071302 9L | 99× | 10,679 | 2,115,840 | 35.7 | 2,138 | 0 |
| M23061903 1.1K | 100× | 10,857 | 2,105,329 | 35.6 | 2,119 | 0 |
| M21110306 7 | 103× | 7,556 | 2,125,773 | 35.7 | 2,144 | 0 |
| M23051104 40c | 101× | 8,193 | 2,112,164 | 35.7 | 2,136 | 0 |
| M22122205 4.1K | 101× | 11,386 | 2,113,332 | 35.7 | 2,133 | 0 |
| M22122204 7.1B | 82× | 5,072 | 2,113,336 | 35.7 | 2,134 | 0 |
| M22090905 12B | 102× | 10,125 | 2,064,658 | 35.7 | 2,082 | 0 |
| M22063003 6.1K | 100× | 9,794 | 2,132,895 | 35.8 | 2,156 | 0 |
| M21110306 19 | 99× | 10,445 | 2,127,094 | 35.8 | 2,147 | 1 |
| M19092402 F8a | 82× | 9,417 | 2,124,327 | 35.8 | 2,149 | 1 |
| M24061702 9L | 103× | 9,467 | 2,115,864 | 35.7 | 2,138 | 0 |
| M15112301 1 | 100× | 11,815 | 2,176,426 | 35.8 | 2,191 | 0 |
| M17081102 24 | 100× | 9,633 | 2,267,872 | 35.9 | 2,287 | 0 |

**Supplemental Table 2.** Publicly available *Streptococcus agalactiae* CPS type Ia genomes analyzed in the present study for comparative bacterial genomics and phylogenetic analyses. The data provided for each isolate includes the isolate name, year of isolation, country of origin, designation as CPS type Ia-2021 or other Ia, NCBI accession, and reference (if available). All isolates originated from tilapia, with two exceptions notated by superscript letters, and were assigned to MLST sequence type 7/clonal complex 7.

| **Isolate Name** | **Year** | **Country of Origin** | **CPS Type** | **NCBI accession** | **Reference** |
| --- | --- | --- | --- | --- | --- |
| GD201008-001 | 2010 | China | Ia | GCF_000299135.1 | Liu et al. ^3^ |
| YM001^a^ | ― | China | Ia | GCF_001190825.1 | Wang et al. ^4^ |
| HN016 | 2010 | China | Ia | GCF_001190805.1 | Wang et al. ^4^ |
| ZQ0910 | 2010 | China | Ia | GCF_011383065.1 | Wang et al. ^5^ |
| FNA07 | 2009 | Thailand | Ia | GCF_000715295.1 | Kayansamruaj et al. ^6^ |
| KKN 3/1 (L) | 2012 | Thailand | Ia | GCF_001592615.1 | Areechon et al. ^7^ |
| FPrA02 | 2010 | Thailand | Ia | GCF_000715315.1 | Kayansamruaj et al. ^6^ |
| ENC06^b^ | 2011 | Thailand | Ia | GCF_000714695.1 | Kayansamruaj et al. ^6^ |
| 3896VN | 2015 | Vietnam | Ia | GCF_003160745.1 | Kayansamruaj et al. ^8^ |
| FBC60 | 2020 | Philippines | Ia | GCF_040201765.1 | Choresca et al. ^9^ |
| WC1535 | 2015 | China | Ia | GCF_001729925.2 | Unpublished |
| 2108 | 2021 | China | Ia-2021 | GCF_029917085.1 | Unpublished |
| 474 | 2023 | Colombia | Ia-2021 | GCF_032669685.1 | ICA and Agrosavia, unpublished |
| 686 | 2023 | Colombia | Ia | GCF_032669785.1 | ICA and Agrosavia, unpublished |
| 352 | 2023 | Colombia | Ia-2021 | GCF_032669765.1 | ICA and Agrosavia, unpublished |
| 677 | 2023 | Colombia | Ia-2021 | GCF_032669725.1 | ICA and Agrosavia, unpublished |
| 724 | 2023 | Colombia | Ia-2021 | GCF_032669745.1 | ICA and Agrosavia, unpublished |

^a^ Isolate YM001 is an attenuated strain derived from isolate HN016

^b^ Isolate EN06 was recovered from water collected from an earthen tilapia pond

**Supplemental Table 3.** Name and sequences of the PCR primers designed in the present study and previously designed by Imperi et al. ^1^ for the multiplex PCR assay to determine the CPS type of *Streptococcus agalactiae* isolates.

|  | **Primer name** | **Sequence (5’ → 3’)** | **Reference** |
| --- | --- | --- | --- |
| 1 | Ia-old-F | ATCAGTTATTCAGCCAAGG | Present study |
| 2 | Ia-old-R2 | AGTCACATCTTCAAACTCAG | Present study |
| 3 | cpsL-F | CAATCCTAAGTATTTTCGGTTCATT | Imperi et al. ^1^ |
| 4 | cpsL-R | TAGGAACATGTTCATTAACATAGC | Imperi et al. ^1^ |
| 5 | cpsG-F | ACATGAACAGCAGTTCAACCGT | Imperi et al. ^1^ |
| 6 | CpsG-R | ATGCTCTCCAAACTGTTCTTGT | Imperi et al. ^1^ |
| 7 | CpsG-2-3-6-R | TCCATCTACATCTTCAATCCAAGC | Imperi et al. ^1^ |
| 8 | cpsJ-2-4-F | CATTTATTGATTCAGACGATTACATTGA | Imperi et al. ^1^ |
| 9 | cpsJ-2-R | CCTCTTTCTCTAAAATATTCCAACC | Imperi et al. ^1^ |
| 10 | cpsJ-Ib-F | GCAATTCTTAACAGAATATTCAGTTG | Imperi et al. ^1^ |
| 11 | cpsJ-Ib-R | GCGTTTCTTTATCACATACTCTTG | Imperi et al. ^1^ |
| 12 | cpsI-Ia-6-7-F | GAATTGATAACTTTTGTGGATTGCGATGA | Imperi et al. ^1^ |
| 13 | cpsI-6-R | CAATTCTGTCGGACTATCCTGATG | Imperi et al. ^1^ |
| 14 | cpsI-7-R | TGTCGCTTCCACACTGAGTGTTGA | Imperi et al. ^1^ |
| 15 | CpsN-5-F | ATGCAACCAAGTGATTATCATGTA | Imperi et al. ^1^ |
| 16 | CpsN-5-R | CTCTTCACTCTTTAGTGTAGGTAT | Imperi et al. ^1^ |
| 17 | CpsJ-8-F | TATTTGGGAGGTAATCAAGAGACA | Imperi et al. ^1^ |
| 18 | CpsJ-8-R | GTTTGGAGCATTCAAGATAACTCT | Imperi et al. ^1^ |
| 19 | cpsJ-4-R | CCTCAGGATATTTACGAATTCTGTA | Imperi et al. ^1^ |
| 20 | cpsI-7-9-F | CTGTAATTGGAGGAATGTGGATCG | Imperi et al. ^1^ |
| 21 | cpsI-9-R | AATCATCTTCATAATTTATCTCCCATT | Imperi et al. ^1^ |

**References**

1. Imperi, M. et al. A multiplex PCR assay for the direct identification of the capsular type (Ia to IX) of *Streptococcus agalactiae*. *J. Microbiol. Methods* **80**, 212-214 (2010).

2. Shoemaker, C., Xu, D., Garcia, J. & LaFrentz, B. R. Capsular typing of *Streptococcus agalactiae* (Lancefield group B streptococci) from fish using multiplex PCR and serotyping. *Bull. Eur. Assoc. Fish Pathol.* **37**, 190-197 (2017).

3. Liu, G., Zhang, W. & Lu, C. Complete genome sequence of *Streptococcus agalactiae* GD201008-001, isolated in China from tilapia with meningoencephalitis. *J. Bacteriol.* **194**, 6653-6653 (2012).

4. Wang, R. et al. Comparative genome analysis identifies two large deletions in the genome of highly-passaged attenuated *Streptococcus agalactiae* strain YM001 compared to the parental pathogenic strain HN016. *BMC Genomics* **16**, 897 (2015).

5. Wang, B. et al. Complete genome sequence of *Streptococcus agalactiae* ZQ0910, a pathogen causing meningoencephalitis in the GIFT strain of Nile tilapia (*Oreochromis niloticus*). *J. Bacteriol.* **194**, 5132-5133 (2012).

6. Kayansamruaj, P., Pirarat, N., Kondo, H., Hirono, I. & Rodkhum, C. Draft genome sequences of *Streptococcus agalactiae* strains isolated from Nile tilapia (*Oreochromis niloticus*) farms in Thailand. *Genome Announc.* **2(6)**, e01300-01314 (2014).

7. Areechon, N., Kannika, K., Hirono, I., Kondo, H. & Unajak, S. Draft genome sequences of *Streptococcus agalactiae* serotype Ia and III isolates from tilapia farms in Thailand. *Genome Announc.* **4(2)**, e00122-00116 (2016).

8. Kayansamruaj, P. et al. Comparative genomics inferred two distinct populations of piscine pathogenic *Streptococcus agalactiae*, serotype Ia ST7 and serotype III ST283, in Thailand and Vietnam. *Genomics* **111**, 1657-1667 (2019).

9. Choresca, C. H. et al. Draft genome of *Streptococcus agalactiae* serotype Ia FBC260 causing hemorrhagic septicemia with massive cellular meningitis in cultured Nile tilapia from the Philippines. *Microbiol. Resour. Announc.* **14**, e00911-00924 (2025).
